# DeePay: deep learning decodes EEG to predict consumer’s willingness to pay for neuromarketing

**DOI:** 10.3389/fnhum.2023.1153413

**Published:** 2023-06-05

**Authors:** Adam Hakim, Itamar Golan, Sharon Yefet, Dino J. Levy

**Affiliations:** ^1^Neuroeconomics and Neuromarketing Lab, Sagol School of Neuroscience, Tel Aviv University, Tel Aviv-Yafo, Israel; ^2^Amir Globerson Research Group, Blavatnik School of Computer Science, Tel Aviv-Yafo, Israel; ^3^Neuroeconomics and Neuromarketing Lab, Coller School of Management, Tel Aviv University, Tel Aviv-Yafo, Israel

**Keywords:** neuromarketing, deep learning, neuroscience, machine learning, electroencephalogram, consumer neuroscience, neural networks, consumer behavior

## Abstract

There is an increasing demand within consumer-neuroscience (or neuromarketing) for objective neural measures to quantify consumers’ subjective valuations and predict responses to marketing campaigns. However, the properties of EEG raise difficulties for these aims: small datasets, high dimensionality, elaborate manual feature extraction, intrinsic noise, and between-subject variations. We aimed to overcome these limitations by combining unique techniques of Deep Learning Networks (DLNs), while providing interpretable results for neuroscientific and decision-making insight. In this study, we developed a DLN to predict subjects’ willingness to pay (WTP) based on their EEG data. In each trial, 213 subjects observed a product’s image, from 72 possible products, and then reported their WTP for the product. The DLN employed EEG recordings from product observation to predict the corresponding reported WTP values. Our results showed 0.276 test root-mean-square-error and 75.09% test accuracy in predicting high vs. low WTP, surpassing other models and a manual feature extraction approach. Network visualizations provided the predictive frequencies of neural activity, their scalp distributions, and critical timepoints, shedding light on the neural mechanisms involved with evaluation. In conclusion, we show that DLNs may be the superior method to perform EEG-based predictions, to the benefit of decision-making researchers and marketing practitioners alike.

## 1. Introduction

In the last decade, the field of consumer neuroscience, or neuromarketing, is flourishing, with numerous publications, academic programs, initiatives, and companies. There is a growing community of scientists conducting studies in the field that converged into multiple meta-analyses and reviews (Ariely and Berns, 2010; Javor et al., 2013; Fortunato et al., 2014; Smidts et al., 2014; Hsu and Yoon, 2015; Plassmann et al., 2015; Schneider and Woolgar, 2015; Karmarkar and Yoon, 2016; Genevsky et al., 2017; Hsu, 2017; Karmarkar and Plassmann, 2017; Lee et al., 2017; Harris et al., 2018; Lin et al., 2018; Cherubino et al., 2019). There is an increased demand for objective neural measures that quantify consumers’ subjective valuations and predict responses to marketing campaigns. Many researchers and practitioners aspire to measure neural and physiological activity to predict a future decision or action of an individual or to assess the success of possible marketing campaigns in the general population. This aspiration is driven by the limitations of traditional marketing techniques, such as questionnaires, focus groups, and interviews (Hakim and Levy, 2019).

The notion that a signal for subjective value could be obtained from neural information is based on the inception of the “value network,” together with its growing body of evidence (Levy and Glimcher, 2012; Bartra et al., 2013; Glimcher and Fehr, 2014). The network is considered to compute information from different origins, be they related to personal experience, emotions, memories, impulses, or responses to contexts, and translate it onto a common neural currency, with which decision-makers may compare disparate alternatives to achieve a resolution. Several distinct brain regions are suspected to participate in this network, mainly the ventral striatum, ventromedial prefrontal cortex (vmPFC), and the posterior cingulate cortex, which were shown to be related to subjective value irrespective of reward type, task, or stage of the decision-making process (Kable and Glimcher, 2007; Plassmann et al., 2007; Chib et al., 2009; Levy and Glimcher, 2011; Padoa-Schioppa, 2011).

One candidate technique for acquiring value-related neural information is Electroencephalography (EEG)–an electrophysiological method that records the electrical activity of the brain by attaching several electrodes (electrical conductors) along the scalp (Haas, 2003). EEG recordings provide an approximation of neurotransmitter-mediated neural activity, with high temporal resolution (milliseconds) but poor spatial resolution (Luck, 2014). This technique is commonly used in the neuromarketing industry (see NMSBA Website), and there is accumulating evidence linking various EEG signals with value-based choice (Sutton and Davidson, 2000; Dmochowski et al., 2012; Fuentemilla et al., 2013; Khushaba et al., 2013; San Martin et al., 2013). Several academic studies have already employed EEG recordings to predict subjects’ stated valuation or actual choices (Vecchiato et al., 2011; Kong et al., 2013; Ravaja et al., 2013; Telpaz et al., 2015; Yadava et al., 2017; Ramsøy et al., 2018; Wei et al., 2018; Golnar-nik et al., 2019; Kumar et al., 2019; Alnuman et al., 2020; Pandey et al., 2020; Si et al., 2020), or population marketing success (Dmochowski et al., 2014; Boksem and Smidts, 2015; Venkatraman et al., 2015; Barnett and Cerf, 2017; Christoforou et al., 2017; Guixeres et al., 2017; Shestyuk et al., 2019; Eijlers et al., 2020). However, only a few tried to use the latest computational modeling techniques. Importantly, previous studies relied on manual feature extraction and had small data sets.

For example, a recent study recorded EEG data from 30 participants as they watched 220 different advertising commercials, and used an support vector machine (SVM) model on manually extracted features to predict participants’ questionnaire responses that capture their purchase intent (Wei et al., 2018). Another study predicted the effectiveness of Super Bowl commercials based on features extracted manually from the EEG signals alongside eye tracking and heart rate data (Guixeres et al., 2017). The authors used a simple neural network with only two fully-connected layers with ten and five units, respectively, and attempted to predict the number of YouTube viewings for each commercial. Additionally, a team from Neuromarketing Labs reported they could predict, using features manually extracted from the EEG signals, between successful and unsuccessful sales of shoes. They used a custom-made “preference index computed through an internally developed algorithm loosely associated with parameters from basic emotional neuroscience” (Baldo et al., 2015). Another study applied a hidden Markov model on features they extracted from the EEG signals of 40 participants while they were watching images of 14 different products, to predict their likes/dislikes of the products (Yadava et al., 2017). The authors also tested various other models, such as support vector machine, random forest, and nearest neighbors.

A different study applied a probabilistic neural network and k-nearest neighbors prediction models (Murugappan and Murugappan, 2014) to predict preferences. In that study, participants viewed four commercials per four different vehicle brands while their EEG activity was measured and processed manually to several features. Lastly, a study conducted in our lab used a combination of a multitude of EEG measures to predict choices that we manually extracted based on previous literature: inter-subject correlations (ISC), hemispheric asymmetry, and spectral power bands (Hakim et al., 2021). Subjects watched six different product commercials while we recorded their EEG activity. We predicted both subjects’ choices and the commercials’ success at the population level. Importantly, we showed that adding the EEG data increased the prediction success compared to what we could achieve when using only a standard marketing questionnaire. To accomplish this, we utilized various machine learning models, such as support vector machines, decision trees, kernel discriminant analysis, and more.

Although these studies demonstrated successful predictions, they all manually extracted the features of the EEG signal. Moreover, each study used a different combination of features as the input to their model. Hence, as a scientific community, this prevents us from converging to well-established and generalizable EEG-based neural signals that represent value across various domains, stimuli, and environments. To overcome these shortcomings, in the current study, we use a state-of-the-art deep learning model both as our predictive algorithm and as our feature extraction procedure.

The clear dogma that emerges from examining the previous EEG-based prediction studies is that they manually extracted various features from the EEG signal and used some classifier to learn the conditional probability of subjective valuations on the extracted features. As shown above, the features extracted rely on previous findings relating these features to different aspects of the valuation process (Hakim and Levy, 2019). We propose that this approach to the prediction task is fundamentally limited and suffers from issues constraining its success.

First, the properties of EEG datasets, in general, raise difficulties for conventional machine learning and regression methods in prediction. This is because the size of the EEG datasets is often small, the EEG signal is high dimensional, very noisy, obscure, non-stationary, and with high unpredictable variations between subjects. Moreover, there is a need for elaborate and exploratory feature extraction.

Second, in all previous attempts, the experimenters chose in advance, based on previous studies, which EEG features to use for their prediction. Thus, researchers limit the information that could be extracted from the EEG recordings to whatever can be captured based on their specific pre-defined pre-processing and feature extraction pipelines. However, there are unlimited possibilities of features to use and pre-processing parameters to tune manually. For example, assume we want to use theta frequency power band (which is in the range of 4−8 Hz) as an input to the model. One question that immediately arises is whether to take the entire range from 4−8 Hz, or maybe just the range between 5−7 Hz, or perhaps allow each subject her unique range. Moreover, researchers must choose various parameters with which to conduct the spectral transformation, such as the time window, overlap, frequency resolution, and transformation technique (wavelet, STFT, etc.). These questions only scratch the surface, as there are endless possibilities and free parameters to decide from what time points to extract any feature. On top of that, we can add the variety of options available for treating specific electrodes and the signal’s spatial components (Averaging, maximizing, ICA, etc.).

There are numerous possibilities in each feature extraction pathway, giving the researcher countless degrees of freedom. It is almost impossible to know in advance which exact feature, pre-processing method, and extraction procedure will give the best prediction. This allows the researcher to explore various procedures and arrive at the most predictive features for his/her dataset, which could easily result in overfitting the dataset and failing to generalize the results on new data. We propose that this might explain the limited success of EEG-based features in out-of-sample predictions. In fMRI, the degrees of freedom during the pre-processing stage were estimated to be in the order of tens of thousands (Poldrack et al., 2017), and we contend that EEG is not different in this matter.

Lastly, and this is mainly a problem for regression approaches, even if we can choose the best possible features without overfitting, we still need to figure out if there are interactions between these features and what exactly they are. For example, it is often unclear whether we need double or triple interactions between each feature in the model, or whether their interaction may be non-linear, and so on.

To overcome these limitations, in this study, we use a Deep Learning Network (DLN) (LeCun et al., 2015) that combines several state-of-the-art techniques. Thanks to their superior ability to learn complex representations and automatically extract optimal features, DLNs have revolutionized many research fields, such as Computer Vision, Brain-Computer Interfaces, Artificial Intelligence, and more. Importantly, DLNs have already shown success utilizing EEG signals for many applications (Khosla et al., 2020; Gu et al., 2021). The applications vary from epilepsy prediction and seizure monitoring (Mirowski et al., 2009; Antoniades et al., 2016; Hosseini et al., 2016; Liang et al., 2016; Page et al., 2016; Thodoroff et al., 2016; Golmohammadi et al., 2017; Acharya et al., 2018), to auditory music retrieval (Stober et al., 2014, 2015), detection of visual-evoked responses (Cecotti and Graser, 2011; Cecotti et al., 2014; Manor and Geva, 2015; Shamwell et al., 2016), mental workload classification (Bashivan et al., 2015; Almogbel et al., 2019), sleep stage recognition (Supratak et al., 2017; Sors et al., 2018; Humayun et al., 2019; Michielli et al., 2019; Yuan et al., 2019), emotion detection (Jirayucharoensak et al., 2014; Zheng and Lu, 2015; Kim and Jo, 2018; Teo et al., 2018; Chen et al., 2019; Qazi et al., 2019; Zeng et al., 2019; Li et al., 2020), limb control (Nakagome et al., 2020), biometric authentication (Jalaly et al., 2020), and motor imagery classification (An et al., 2014; Li and Cichocki, 2014; Sakhavi et al., 2015; Sakhavi and Guan, 2017; Schirrmeister et al., 2017; Tabar and Halici, 2017; Tang et al., 2017; Olivas-Padilla and Chacon-Murguia, 2019; Qiao and Bi, 2019; Tayeb et al., 2019). Moreover, a review of 154 papers found that the median gain in accuracy of DLN approaches compared to regression and other machine learning models was 5.4% (Roy et al., 2019).

In our study, we developed a novel DLN architecture that decodes EEG neural signals to predict willingness to pay (WTP) for products. Since DLNs require a large amount of data, we collected an extensive dataset that has over 35k samples of EEG recordings from 213 subjects. We acquired the EEG recordings while subjects observed pictures of products and then reported their WTP for the observed product, as a proxy for their individual subjective values. This paradigm could later translate to prediction of subjective valuations of products in response to marketing stimuli, although further research would be required to establish this. Then, we applied a DLN to overcome the drawbacks of EEG measurements stated above and optimally performed predictions of subject-specific WTP. We combined several state-of-the-art techniques that alleviate the need to manually search for features in the EEG signal that contain value-related information, without *a priori* choosing any features or preprocessing pipelines. Lastly, we used network visualization techniques to gain insights regarding the attributes of the signal that best predict value and thus the neural mechanisms involved.

## 2. Materials and methods

### 2.1. Subjects

To our knowledge, none of the brain computer interface (BCI), EEG, or Neuromarketing communities have published a dataset with EEG recordings and corresponding continuous valuations of products in the scale appropriate for deep learning. That is, a dataset that would include both a large sample size and multiple trials per participant. Although, a relevant study was conducted by Sundararajan et al. (2017) who collected a total of 1,800 samples of EEG recordings from 181 subjects, albeit using only binary choices between products and not a continuous value scale such as WTP. Therefore, we have undertaken the task of creating such a dataset in our own laboratory. Two hundred and thirteen subjects (96 males) participated in the study, aged 19−51 (Mean = 25.36, STD = 5.07). We excluded 30 subjects from the analysis based on our strict exclusion criteria (elaborated later) and a single recording failure, resulting in a total of 183 subjects. All subjects gave written informed consent before participating in the study, which was approved by the local ethics committee at our university.

### 2.2. Stimuli

Stimuli consisted of 72 pictures of products from 6 different categories (12 products per category). In addition to the picture of the product, there was a short text above the picture describing the product. See Appendix A for a full list of products. Products were chosen such that their real-world cost exceeded the maximal bid amount, incentivizing subjects to bid for the products regardless of any cost-benefit calculation related to their real-world value, but rather to reflect their subjective value. Their market price ranged from 45 to 99 New Israeli Shekel (NIS, Mean = 69.23, STD = 16.05).

### 2.3. Experimental design

For each subject, we first presented the full experimental instructions and applied a short verbal test to verify they understood the task. They received 50 NIS as a participation fee and an additional 50 NIS as the endowment for the behavioral task. Subjects were instructed to sit upright in front of the computer screen, maintain their position with minimal movements throughout the task.

Then, we mounted them with an 8-electrode (wet) EEG system (StartStim 8 system by Neuroelectrics, Spain) at positions F7, Fp1, Fpz, Fp2, F8, Fz, Cz, Pz, sampled at 500 Hz. We opted for a lean and cost-effective 8 (wet)-electrode array to increase the applicability of our study and to demonstrate that lean arrays can be effective and reliable as neural-based value prediction tools. Additionally, we focused mainly on the frontal electrodes because they are easy to apply, require less gel, and are present in most simple headsets used in the industry. Moreover, they have been shown to be related to value representations in many previous studies (Braeutigam et al., 2004; Smith and Gevins, 2004; Khushaba et al., 2012, 2013; Koelstra et al., 2012; Ravaja et al., 2013; Luck, 2014; Boksem and Smidts, 2015; Telpaz et al., 2015; Yadava et al., 2017). Lastly, mounting a dense EEG device on multiple subjects could be strenuous for researchers and practitioners, in terms of costs, effort and discomfort. The extra effort may often not yield additional predictive value, as electrodes that are physically close to each other are highly correlated, on the order of *r* = 0.8−0.98 (Bhavsar et al., 2018). Nevertheless, we agree that using more electrodes while covering additional scalp locations might benefited our models’ predictions.

**FIGURE 1:**
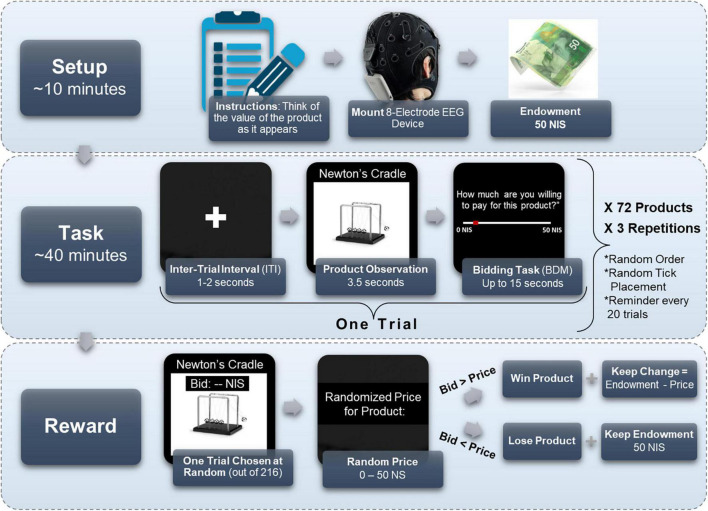
Experimental design. Subjects first received instructions, were mounted with an 8-electrode EEG cap, and received their 50 NIS endowment. Then, they performed the BDM task, for 72 different products, each repeating three times in a random order for a total of 216 trials. On each trial, subjects watched a screen with a plus icon for 1–2 s, then observed the product for 3.5 s, and finally had 15 s to mark how much they were willing to pay for the observed product, from 0 to 50 NIS. Importantly, the starting point of the tick marker on the bid scale (in red) was randomly located in each trial. Every 20 trials a reminder screen appeared to remind subjects to think on their bid as they observe a product. At the end of the task, one trial was chosen at random, and a random price was given to the product at that trial. If the bid was larger than the price, the subject won the product and the remainder of the endowment (50 NIS minus the price of the product). If the bid was lower than the price, the subject did not win the product, but kept the full endowment.

As can be seen in Figure 1, on each trial, while subjects watched each product on the computer screen, they were instructed to think how much they value the product. Each product was presented for 3.5 s. Afterward, a horizontal sliding bar appeared, and subjects stated their maximal amount of money they were willing to pay for the product (WTP), between 0 and 50 NIS (∼14.5$). Subjects had up to 15 s to state their WTP. The sliding bar was accompanied by the instruction, “Please indicate how much you would be willing to pay for the product.” When a subject understands the task correctly and the procedure, their WTP bid should correspond to her subjective value for the product.

Importantly, we randomly initialized the location of the “tick” that indicates the chosen amount on the sliding bar, such that subjects did not know in advance whether and how far they would be moving the cursor left or right on the sliding bar after seeing the product. We did this to avoid motor preparation signals in the EEG recordings, which could be correlated with value and obscure our desired value signal. Lastly, a black screen appeared as an inter-trial-interval (ITI) for 1 to 2 s (duration chosen at random uniformly), and the subsequent trial would commence. See Figure 1 for an illustration of the experimental procedure. Every 20 trials, a screen appeared with the instruction “Remember that you must think of the value of the product as it appears before you,” which required a mouse click to resume. This screen served as a reminder for subjects to think of the value of the products as they observe them, in hopes that it strengthened the value signal in the EEG recordings.

Subjects viewed each product 3 times for a total of 216 trials in random order. The three repetitions were intended to expand our dataset by obtaining more samples of the same product from each of our subjects, without overextending the experiment or overcomplicating logistics with additional products. This also enabled the model to reduce noise unrelated to the value signal of a subject’s product viewing. Lastly, it could reveal severe inconsistencies in valuations between blocks, which would point to subject’s disengagement from the task and warrant their exclusion.

We applied the standard Becker-DeGroot-Marschak (BDM) procedure (Becker et al., 1964), a widely popular mechanism to elicit subjects’ WTP (Mejía-Barbosa and Malacara-Hernández, 2001; Noussair et al., 2004; Miller et al., 2011), in order to make the experiment incentive compatible and to obtain precise estimations of subjects’ valuations of the products. That is, after the experiment was finished, one trial was chosen at random. The computer randomly defined a price for the product of the chosen trial, between 0 to 50 NIS (the size of the endowment). If the amount the subject offered for the product in that trial was larger than the randomly defined price–then the subject received the product at the random price and kept the remaining change from her endowment. A subject’s total winnings, in this case, were the product, the 50 NIS participation fee, and the difference between the 50 NIS endowment and the random price. If the amount the subject offered was lower than the randomly defined price generated, then the subject did not win the product and kept the endowment in its entirety, making his total earnings 100 NIS.

This mechanism incentivizes participants to report their most precise estimation, in the given scale, of their subjective value for the product. A subject should not bid higher than her subjective value for a product, as to not pay for it excessively had the randomly defined price surpassed her subjective value toward the product. Also, the subject should not bid lower than her subjective valuation for the product, so she would not lose the opportunity to purchase the product if the randomly defined price were higher than her bid, but lower than or equal to her valuation. In summary, the structure of the task incentivizes participants to bid their best estimation of their subjective value for the product, regardless of its real-world cost (which is irrelevant) or its ensuing price (which is not yet known), in an arbitrary scale from zero to the size of the endowment. Therefore, the value of each bid on the scale, the WTP value, can be viewed as a measure of subjects’ own subjective value for the product.

To reduce any chance of confusion, and to make sure subjects understood the mechanism correctly and report their WTP values according to their subjective value, we explained all these points thoroughly in the instructions before each run, both in text and verbally. In addition, we explicitly stated in the instructions that they are not required to estimate the products’ prices, but to report their WTP for them regardless the products’ real-world prices, since the actual product price would be randomly defined between 0 and 50 NIS. Also, we tested subjects with several simulated scenarios to verify that they understood the mechanism. Lastly, we explicitly asked subjects in every screen prompt on each trial, just above the WTP scale, to indicate how much they would be willing to pay for the product.

### 2.4. EEG preprocessing

The EEG recordings were down-sampled by 2 (from 500 hertz to 250 hertz) and divided to separate epochs, such that each recording during a product observation served as a single sample in the dataset. Down sampling is not mandatory, but recommended for more compact networks, as long as it does not harm accessing the relevant frequency range. An epoch included 3.5 s of product observation and 1 s from the previous ITI, which the model used as the baseline. We discarded the recordings while subjects stated their WTP values, so we would not confound a signal for subjective value with motor execution. The EEG sensors could pick up the cortical activity related to moving the mouse cursor to a selected WTP value, so removing this motor information from the data assured that our model would only identify value-related information. Thus, a sample consisted of 8 channels and 1,125 time points (4.5 s sampled at 250 hertz).

We wanted to minimize any manual pre-processing and filtering, so we could provide the model with the closest data to a raw signal and have the model perform any pre-processing within it. Hence, the only pre-processing steps we conducted was first, to apply a bandpass filter on the whole experiments’ recording of each electrode separately (before dividing into epochs), between 0.5 hertz and 100 hertz (second-order Butterworth), and second, we subtracted the mean signal per sample per electrode. Applying the bandpass infused the model with domain knowledge, namely, that the relevant frequency bands to search for are within this band’s range. Subtracting the average is common normalization practice so gradients do not go out of control. Moreover, we defined criteria to exclude samples with excessive noise. For a given sample, any electrode recording with a standard deviation higher than 50 mV or with a maximal amplitude higher than 400 mV was discarded and replaced by a neighboring electrode (Delorme and Makeig, 2004). If a given sample had four or more electrodes, which correspond with these exclusion criteria, then the entire sample was discarded. Out of all samples from the relevant subjects, 7.37% of the samples were removed following these criteria. One subject was removed due to a failure in recording.

Aside from preparing the EEG data for the network, we also wanted to inspect the data outside of the network, so we created spectrograms of each sample. We transformed each trial’s EEG signal recorded from the FPZ electrode to a spectrogram, using MATLAB’s “spectrogram” function. We used a window size of 1.5 s, with 0.01 s overlap between windows, and inspected frequencies between 0.5 Hz to 70 Hz (with a 0.5 Hz interval). The result was the Power Spectral Density [W/Hz] for every time window and frequency, also termed a “spectrogram,” for each trial.

### 2.5. Behavioral preprocessing

Foremost, we divided all WTP values by 50 (the maximal amount) to normalize them to the range between 0 to 1. Each WTP value served as the label for the EEG recordings of its corresponding product observation. Thus, we attempted to learn and predict these WTP values based on the EEG recordings as specified in the previous section.

**FIGURE 2:**
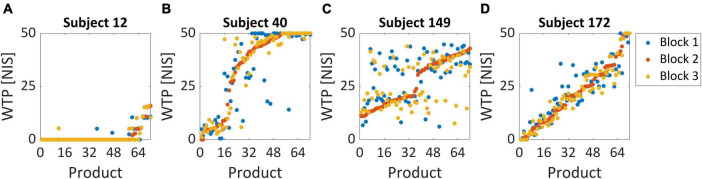
Behavioral exclusion criteria. These graphs demonstrate the WTP values for all three blocks, sorted by the second block (lowest to highest value), for four example subjects. **(A)** Subject 12 was excluded since she bid less than 5 NIS over 85% of her trials, representing very low variability in WTP across products. We excluded 27 subjects based on this criterion. **(B)** Subject 40 was not excluded and is considered a good subject. Note that she bid above 45 NIS in only 47% of her trials and was therefore not excluded. No subjects were excluded based on this criterion. **(C)** For subject 149, the correlation between the second block and third block was 0.41, representing very high variability in WTP values. This might suggest that this subject did not report its “true” valuations but answered randomly or that her actual valuations change very quickly on a block-by-block basis. Two subjects were excluded based on this criterion. **(D)** Subject 172 exhibited consistent behavior across the three repetitions of the BDM blocks and, in addition, highly varied valuation across products. Her correlation coefficient between blocks 2 and 3 was 0.97, her bid rate below 5 NIS was only 12%, and her bid rate above 45 NIS was only 8%.

We enforced several behavioral exclusion criteria, according to subjects’ WTP values, in order to ensure that the task was able to elicit genuine subjective values in our subject pool. Including subjects that did not perform the task seriously or sincerely would damage our dataset and hinder our model’s ability to identify the relationship between the neural signal and actual individual value. If a given subject provided WTP values smaller than 5 NIS (10% of the maximal amount) for more than half of her trials, the subject was excluded entirely from the dataset (Figure 2A). This type of subject had demonstrated that she was disinterested in most of the products offered in the task and therefore could not provide a reliable value signal in her recordings. Additionally, we also tested whether a given subject exceeded 45 NIS (90% of the maximal amount) for more than half of her trials (Figure 2B). This subject would likely be over-enthusiastic to purchase any item without weighing their subjective value properly.

Additionally, we wanted to examine the consistency of valuations between the three different blocks to estimate whether subjects reported persistent values for each item they saw. So, we tested the correlation between the WTP values for the same items in all three blocks. In the exclusion criterion for this analysis, we used only the correlation between the second and third blocks to exclude subjects. This was because we estimated that these correlations should be the highest across our sample since subjects should converge to a constant valuation of a product the more times they evaluate it. Therefore, a subject with an exceptionally low correlation between her second and third blocks was more likely to be a candidate for exclusion. We excluded subjects with a correlation coefficient lower than 0.5 (Figure 2C) since they seemed far apart in their coefficient compared with the rest of the subjects, which at least arrived at 0.74 (Figure 4). These subjects were too inconsistent in their valuations, raising suspicion that their reported valuations were more random. For comparison, an ideal subject would exhibit consistent behavior across the three repetitions of the BDM blocks and have highly varied valuation across products, with a high correlation between the second and third block, and low bid rate below 5 NIS and above 45 NIS (Figure 2D).

Twenty-nine subjects failed in at least one of these criteria, bringing our subject pool to a total of 183 subjects. For all these excluded subjects, the task failed to elicit varied WTP values for the products presented, so their exclusion does not reflect any shortcomings of the DLN and its predictions.

Moreover, before fitting a neural network to the data, we wanted to examine the general behavior in the BDM task and whether valuations of each product varied between subjects, and if subjects were consistent with their valuation of a product across blocks. It is problematic if all subjects have similar WTP values across all products since then predicting a product’s value could be a matter of identifying its image’s particular pattern of neural response, rather than basing the prediction on the subjective value signal it elicited for each subject. If this was the case, the model could hypothetically use the similar WTP values across subjects at the product’s identifier and employ shared neural responses to the product for its prediction instead of predicting each subject’s unique valuation. We addressed this possible problem, in part, by including 72 products in the experiment. This makes it very difficult for a model to hypothetically learn 72 unique product identifiers based on common neural responses to products but instead makes it more likely for the model to learn neural value representation. However, to even further negate the possibly that the network learned unique product identifiers based on common neural responses to products, we inspected how much the WTP values varied between subjects. So, per product, we observed the averaged valuations of products across all subjects and blocks.

### 2.6. Network architecture

We propose a proprietary DLN architecture that considers the unique characteristics of the EEG signal and offers several types of processes and components that account for the signals’ spectral, spatial, temporal, and subject-specific dependencies. This unique architecture accepts the raw EEG signal as input, implements classic signal processing procedures within the network to extract features automatically, provides interpretability throughout these steps, and finally outputs a prediction of WTP. In the first layer, bandpass filters with trainable cuttoff frequencies are applied to the raw EEG multi-channel signal. These create unique “spectral maps” per filter. Then, a spatial convolution is performed on maps from each band separately, accounting for the information from different electrode channels independently for each band. Afterward, the high-dimensional information from the spatial maps is integrated using an economical and parameter-cheap technique, resulting in time-series feature maps. These are fed into recurrent layers and an attention mechanism that captures the relationships between the different temporal sections of the representations extracted from the previous layers, accounting for time-dependencies. Finally, we apply a SoftMax activation function on the final two nodes of the network for the probability of that sample to belong to each class (= High/Low WTP), or we directly output a final node as a single regression output. As meta procedures, we optimized our hypermeters using the Parzen Estimator approach (TPE) (Bergstra et al., 2011) and used an ensemble of networks with random initializations for each prediction. Unfortunately, as there is a patent pending on the architecture of the DLN, we cannot reveal its full details in this paper. However, these will be made available upon request.

### 2.7. Training and evaluation

We conducted several predictions to address our main aims. We wanted to test the ability of the network to conduct both binary classification and continuous predictions (by simply removing the final Soft Max layer in the network). First, we divided the dataset into 80% training, 10% validation, and 10% testing. In our main analysis the split is random across the entire dataset, but we delve into additional types of splits in the next section. The validation set was used for the hyperparameter tuning (TPE) procedure and for early stopping during training.

For training the network in all binary prediction attempts, we took the bottom 0−35% of WTP values and labeled them as class 0, while the top 65−100% was labeled as 1. There was no specific reason for choosing these precise cutoff thresholds, other than to remove the middle third of the data (35−65% of WTP values), and to avoid over fitting these thresholds by selecting them after observing the results. For training the network for continuous value predictions, we used the same data but with the original WTP values. The rest of the samples in the training set were discarded (35−65% WTP). This was done to boost the network’s ability to identify distinct value information while learning, since the 35−65% of WTP values are the closest in value terms, and hence, would be the hardest between which to distinguish. This was also reflected in another analysis we did, where the two mid-ranged quartiles were much more similar to one another than the two quartiles further apart (see Figures 8, 9 below).

For all binary predictions, we report the area under the curve (AUC) score and the accuracy score, while for the continuous predictions, we report the root mean square error (RMSE) score. We report prediction scores that are based on the 10% held-out test set that was randomly pooled out from the entire dataset before training. We conducted several binary predictions, wherein each prediction attempt, the test set was divided based on different WTP percentiles. That is, we report accuracy, AUC, and RMSE on prediction of the top and bottom 20% WTP values (0−20%/80−100%), then on the following 15% (20−35%/65−80%), then the middle 15% (35−50%/50−65%), and finally the overall scores for the entire range of WTP values within the test set. We segmented the reported results in this fashion to provide a better understanding of the model’s performance across diverse levels of difficulties in prediction, instead of only providing result metrics over all the data. We would expect that prediction between the bottom and top 20% WTP values would be easiest and therefore more accurate, as the neural representation would be more distinct. This is in line with the notion of “neural distance,” as detailed in our previous study (Levy et al., 2011; Telpaz et al., 2015; Hakim et al., 2021). The most adjacent 15% of WTP values would be, therefore, harder to predict. For clarification, all reported prediction scores were based on the same single model that was trained on the bottom 0−35% and the top 65−100% WTP values out of the training set, for either binary or continuous predictions.

In order to test the general success of the prediction results of our network, we compared them to the prediction results of several state-of-the-art benchmark techniques and other more standard approaches. First, we shuffled the labels with respect to the samples to obtain a baseline “random” prediction. Next, we manually extracted the same features from the EEG data and used the same procedure as in our previous study (Hakim et al., 2021). Namely, we extracted the inter-subject correlation (ISC), the spectral power, and the hemispheric asymmetry for all five standard frequency bands (Delta, Theta, Alpha, Beta, Gamma). Additionally, we extracted the three ERP components we identified in the current study (see results of our ERP analysis below) to increase the prediction capabilities of these manually extracted features. We used XGBoost (Chen and Guestrin, 2016), SVM, and Linear/Logistic Regression (depending on whether it was a binary or a continuous value prediction) as the machine learning models. Out of the three models we tested, XGBoost scored the highest, and therefore we present it here and compare it to the performance of our network. Additionally, in order to boost this approach as best as we could, we attempted several feature selection techniques. Namely, we tried PCA, Stepwise elimination, and importance thresholding using XGBoost’s feature importance. Using the importance thresholding outperformed the other two approaches, so we present its results in Table 1. In this technique, we first train the model on the entire set of features to obtain their importance via XGBoost’s internal importance scoring technique. Then, we sort features by their importance, and train and evaluate the model on an increasing number of features, from highest to lowest importance, until the optimal accuracy is obtained. This resulted in excluding 30% of features compared to the naïve model. We believe that the manual feature extraction approach we used alongside the machine learning model was a legitimate contestant to the automatic feature extraction performed via our deep learning network. We think that this comparison can test if our claim regarding the superiority of the DLN framework in prediction holds.

**TABLE 1:** Prediction results.

WTP quantiles	0−20%/80−100%			20−35%/65−80%			35−50%/50−65%			Total		
**Score**	**Acc**	**AUC**	**RMSE**	**Acc**	**AUC**	**RMSE**	**Acc**	**AUC**	**RMSE**	**Acc**	**AUC**	**RMSE**
DL: DeePay	**80.71%**	**0.894**	**0.316**	**67.46%**	**0.738**	**0.211**	55.75%	0.587	**0.194**	**75.09%**	**0.832**	**0.276**
DL: EEG-TCNet	76.24%	0.867	0.348	64.81%	0.716	0.235	**57.13%**	**0.617**	0.205	71.82%	0.804	0.307
DL: Amin et al., 2019	74.53%	0.845	0.372	65.48%	0.721	0.243	56.17%	0.608	0.231	70.18%	0.768	0.319
DL: EEGNet	72.77%	0.798	0.403	60.93%	0.649	0.342	56.81%	0.593	0.263	67.67%	0.737	0.342
DL: Khare and Bajaj, 2021	72.96%	0.832	0.382	62.36%	0.671	0.286	55.46%	0.576	0.275	68.54%	0.743	0.358
DL: DeepCovNet	73.58%	0.828	0.397	60.71%	0.660	0.358	54.44%	0.568	0.280	68.13%	0.761	0.353
DL: ShallowCovNet	73.74%	0.820	0.389	59.41%	0.632	0.363	54.08%	0.564	0.291	67.82%	0.746	0.348
ML: XGBoost–FS	67.13%	0.728	0.421	62.84%	0.648	0.351	56.01%	0.570	0.281	62.25%	0.673	0.365
ML: XGBoost	65.57%	0.713	0.443	61.24%	0.652	0.350	55.17%	0.573	0.286	60.73%	0.659	0.382
ML: Regression (logistic/Linear)	63.72%	0.694	0.481	60.33%	0.618	0.359	53.82%	0.546	0.304	59.13%	0.635	0.406
DL: DeePay (shuffled)	52.32%	0.49	0.512	50.09%	0.491	0.437	54.12%	0.523	0.326	51.82%	0.51	0.447

Accuracy, area under the curve (AUC), and root mean square error (RMSE) for seven different prediction procedures/models, as detailed in the left-most column. We report each of these scores for four different subsets of the dataset–the first and last 20% of WTP values in the data (0−20%/80−100%), the next 15% of WTP values from the top and bottom of data (20−35%/65−80%), the following 15% that represent the center of WTP values (35−50%/50−65%), and finally the results on the entire dataset. Results in bold mark the best score in each column.

We also added two well-known and often used deep learning models–DeepCovNet (Schirrmeister et al., 2017) and EEGNet (Lawhern et al., 2018). The first is a standard convolutional neural network, and the second is a popular network with architecture specialized for EEG decoding and prediction. Moreover, to stand on par with the latest available state of the art networks, we implemented and compared our results to three additional deep learning networks (Amin et al., 2019; Ingolfsson et al., 2020; Khare and Bajaj, 2021). We optimized all the additional models using the same TPE procedure we used for our network.

Lastly, we performed a hyperparameter tuning procedure to set various parameters of our network. The TPE process was evaluated with a validation dataset consisting of 10% of the data and provided the hyperparameters we used for the final predictions on the test set. These parameters include kernel sizes, number of filters, dropout probability, learning rate, batch size, and more. The dropout probability was set to 0.23 throughout the network, the learning rate was set to 0.001, and we used the standard “Adam” optimizer.

### 2.8. Leave-out procedure

An important aspect that must be examined is the generalizability of the network’s architecture, whether it could successfully predict when dividing the data into train and test in various ways. Therefore, in addition to our main prediction results, we examined how our model performs compared to the best alternative model frameworks in different generalization problems. In all the following analyses, we conducted the same procedures for producing results as in the previous section, but for brevity, we report only the prediction results on the entire test set, without examining different quantiles.

First, we split the data to the train and test sets based on subjects’ IDs (1-Subject-Out). That is, there was no data from the same subject in both the train and test sets. We conducted this split, training and evaluation procedure multiple times, each time leaving a single subject in the test set and train on the other subjects, until we tested all our subjects. This way, we could examine our model’s ability to generalize over subjects, which would be necessary when using the model to predict on a new subject. However, since training on all subjects but one resulted in very small test sets, we conducted the same analysis but left 20 subjects out in the test set on each split, which account for nearly 10% of the data (20-Subjects-Out).

Next, we wanted to inspect how well our model generalized over products (12-Products-Out) and product-categories (1-Category-Out). This analysis was important to show that our model could predict WTP competitively even on products or product-categories that it had never seen before. This would reflect cases where the model is used to predict on new products, but using some existing data on the subject from previous products (i.e., model-tuning on a subject). So, using the same procedures, we split the data according to the products, such that in each split, data belonging to a different set of random 12 products was left out in the test set. This provided us with six different test sets that included 12 different random products each (out of 72). To further increase robustness, we tested three additional randomizations of this 6fold split. Additionally, we used the same splitting and testing procedure on a product-category basis, such that in each split a different set of 12 products, belonging to the same product-category, were left out of training. That is, the model evaluation was conducted on products from a single category on each split, while the model was trained on all other categories.

Finally, we combined the 20-Subject-Out with the 1-Category-Out procedure, leaving 1 category in each test set (a 6fold split), while also preserving 20 random subjects entirely in the test set (three randomizations in each fold). This procedure is more applicable in the real world and provides more generalizability since it reflects a scenario where the model must perform a prediction on a product and a subject it has never seen before.

### 2.9. Interpreting the network

Deep learning models are typically regarded as black boxes. Although these models reach impressive prediction accuracies, their nested non-linear structure makes them highly non-transparent. That is, it is not clear what information in the input data makes the model arrive at its predictions (Samek et al., 2017, 2019; Dosilovic et al., 2018; Xie et al., 2020). The use of deep neural networks (DNNs) in neuroscience has created a heated debate about their scientific value and has spurred a discussion whether they are only valuable as predictive tools or might also offer helpful explanations of phenomena (Cichy and Kaiser, 2019). Cichy and Kaiser establish three different perspectives from which DNNs have explanatory power: (i) they provide teleological explanations; (ii) despite their deceptive appearance, they provide the same explanations as traditional mathematical theoretical models; and (iii) owing to their complexity, they have strong potential for *post hoc* explanations.

Therefore, in addition to our DLN’s ability to decode EEG data optimally and adequately, we designed the structure of the layers and their resulting weights in such a way that after the model learned the training data, we can gain insights into what the model deemed most predictive. Thus, we believe that the deliberate design of our layers allowed us to interpret the model’s output in line with Cichy’s and Kaiser’s arguments. The spectral filters embedded in our DLN provide insights into the neural frequencies most predictive of value. The spatial filters demonstrate the spatial distribution of the importance of each frequency, and the attention layers provide information regarding the critical time points for prediction. All these increase our understanding of the neural representation of value and its prediction in general.

### 2.10. Standard EEG analysis

Inspecting Event-Related Potentials (ERP) is a common technique when analyzing EEG signals. In ERP analysis, we average the EEG recordings across different trials of each condition and examine if there are differences in the averaged electrical potentials between conditions. Differing neural responses could shed light on the neural mechanism that is at work in relation to the experimental manipulation between conditions. Therefore, after conducting the primary analysis using our DLN, we conducted several standard ERP-based analyses.

In a typical ERP design, there are multiple repetitions in each experimental condition in order to average out the noise in the signal. However, our experimental design was not a classic ERP design, as there were only three repetitions for each of the 72 products. Therefore, to have a better chance to observe distinct EEG components, and to gain more power in the statistical analysis, we created discrete conditions with a higher number of trials in each condition and averaged them, in order to clean the noise from the signal. So, we divided the data into four quartiles (serving as conditions) based on the WTP values. That is, we created four groups that correspond to the bottom 25% WTP values, 25−50%, 50−75%, and 75−100% of WTP values. We then averaged all the EEG activity from electrode FPZ for trials from each quartile across subjects and examined the signal within a 4.5-s time window (3.5 s of product presentation plus 1 s from the previous ITI). We chose to examine the three frontal electrodes because we previously showed that they carry value-related information (Hakim et al., 2021). Moreover, because the correlations across the three frontal electrodes were very high (*r* = 0.8–0.95 across all subjects), it was enough to focus on the middle electrode, FPZ, as a representative for frontal activity.

In addition, the components derived from the event-related potentials often do not reveal the complete story, as they only expose task-relevant fluctuations in the time domain. Therefore, another popular technique for analyzing the EEG signal is to use spectral decomposition, which adds another perspective of analysis. Thus, we conducted a spectral analysis (as detailed in the Section “2. Materials and methods”) and arranged the spectrograms according to the WTP quartiles. As a first-order analysis, similar to what we have shown in our previous studies (Telpaz et al., 2015; Hakim et al., 2021), we wanted to examine if there is a difference between the spectrograms of the top and bottom WTP quartiles and whether this difference was more pronounced than the difference between the two middle quartiles. Thus, we averaged the spectrogram within each quartile and subtracted the top quartile average spectrogram from the bottom quartile average (Figure 9A), and from the third quartile average spectrogram, we subtracted the second quartile’s average spectrogram (Figure 9B).

## 3. Results

### 3.1. Behavioral results

**FIGURE 3:**
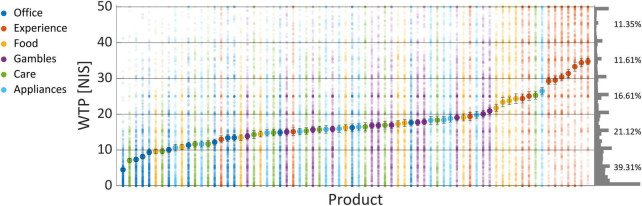
WTP values per product. The above shows the average WTP values across subjects and blocks as “filled” large dots, and the actual values scattered around them as semi-transparent smaller dots, so a more popular value for a product will appear as more saturated and denser dots. The right of the graph shows the frequency of each WTP value across all products and subjects, binned to a width of 1 NIS. The percentages signify the propensity of all WTP values binned to a width of 10 NIS each, from 0 to 50. Error bars signify the standard error of the mean (SEM).

As can be seen in Figure 3, there was a gradual increase in WTP across products averaged over subjects. Products from the “Experience” category were the most preferred products, as six products from this category had the highest WTPs. In the bottom end, the “Office” category was most prevalent, with seven products from that category at the bottom 15 least preferred products. Notably, the WTP values spread around the average of each product, such that no value could be said to correspond to a singular product uniquely. To further substantiate this claim, we tried to predict High/Low WTP values (using the same procedure as in the Section “3.2.1. WTP prediction”), based on the item name and its category, coded as dummy variables (or one-hot vectors). If product identity did not predict WTP values, then it would be highly improbable that EEG-based prediction of WTP was mediated by product identity. We reached 58% prediction accuracy using logistic regression, showing that the identity of products could predict WTP values to a small degree, but not nearly enough to explain the EEG-based predictions of WTP that we demonstrated with our model in the following subsections.

Regardless of this issue, we could also see that subjects seemed to prefer bidding rounded values, with zero and 50 being most prominent, but also 5, 10, 15, and so on. This can be seen on the histogram chart that appears on the right of Figure 3, which shows the relative frequency of each WTP value across all products and subjects.

**FIGURE 4:**
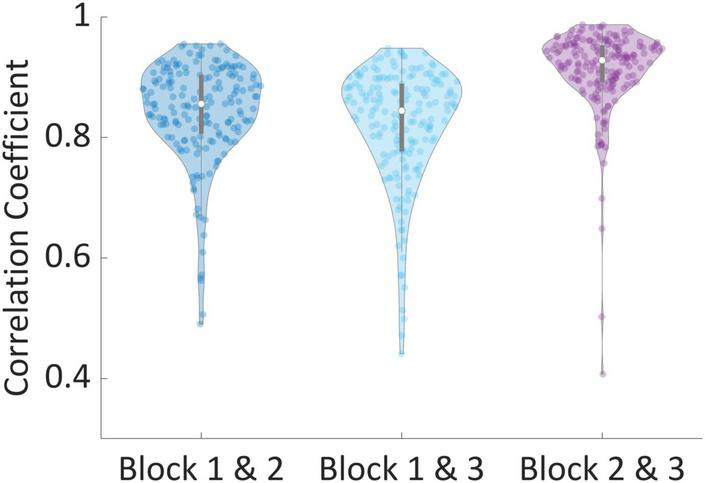
Correlation of valuations between blocks. The correlation between valuations of the same products between every two blocks, per subject. Correlations between blocks 2 and 3 were significantly larger than correlations between blocks 1 and 2 [*t*(181) = 8.39, *p* < 0.001] and blocks 1 and 3 [*t*(181) = 10.15, *p* < 0.001].

Next, for each subject, we calculated three Pearson correlation coefficients across all the valuations of products; Between their valuations in the first and second blocks (Mean = 0.845, STD = 0.087), between the first and third blocks (Mean = 0.821, STD = 0.096), and between the second and third blocks (Mean = 0.911, STD = 0.072). We found that 99% of all correlations for all subjects were above 0.5, while 90% of correlations were above 0.75, and 38% of all correlations were above 0.9. This demonstrates that subjects were highly consistent in their valuations across blocks. To corroborate our approach to exclusion of subjects using the correlations between the second and third block, we performed one-way dependent *t*-tests, hypothesizing that subjects’ correlation between the second and third blocks are higher than the other correlations. Indeed, correlations between block 2 and 3 were significantly larger than correlations between blocks 1 and 2 [*t*(181) = 8.39, *p* < 0.001] and blocks 1 and 3 [*t*(181) = 10.15, *p* < 0.001], as can be seen in Figure 4.

### 3.2. Prediction results

In this subsection we provided all prediction results as described in the Section “2. Materials and methods.” These include WTP prediction, both binary and continuous, from our network as well as other state-of-the-art networks and machine learning approaches. Additionally, we show results for the leave-out procedures.

#### 3.2.1. WTP prediction

Results in Table 1 show that our DLN, termed DeePay, achieved better prediction scores compared to the other DLNs, the classic machine learning model using manual feature extraction with or without feature selection, and the model trained on shuffled labels. Only when trying to differentiate between the percentiles closest to each other and to the median (i.e., a binary classification between 35−50% and 50−65%), our DLN framework did not outperform other models, as differentiating between such close WTP values was a difficult challenge for any model. Indeed, no model achieved significant results for this slight percentile difference, and their prediction accuracies were most likely obtained by chance, yielding no superior model. For a more elaborate evaluation of our main test results, the prediction of low (0−20%) versus high (80−100%) quantiles with DeePay, including the confusion matrix, precision, recall, and F1 scores, see Appendix E. We will gladly provide the same elaboration for any of our other prediction attempts upon request.

#### 3.2.2. Prediction generalizability

Results from all leave-out procedures described in the Section “2. Materials and methods” are available in Table 2. In the first column, we show the average results over all 1-subject-out splits, while in the second column we show the results for the 20-subject-out procedure. Next, the average results over all splits for the 12-product-out procedure are presented in the third column, and the average results from the 1-category-out procedure are described in the fourth column. Finally, the last column shows results for the combined 1-category-out and 20-subject-out analysis. As can be seen in Table 2, our network successfully generalized over subjects, products, and categories. Importantly, our network still exhibited impressive prediction accuracies over these generalization tests and scored better than other DLNs and feature extraction-based frameworks.

**TABLE 2:** Generalization results.

Leave-out procedure	1-Subject-out			20-Subjects-out			12-Products-out			1-Category-out			Subject and category-out		
**Score**	**Acc**	**AUC**	**RMSE**	**Acc**	**AUC**	**RMSE**	**Acc**	**AUC**	**RMSE**	**Acc**	**AUC**	**RMSE**	**Acc**	**AUC**	**RMSE**
DL: DeePay	**74.14%**	**0.830**	**0.253**	**69.41%**	**0.764**	**0.346**	**72.82%**	**0.797**	**0.291**	**71.44%**	**0.783**	**0.315**	**69.29%**	**0.755**	**0.349**
DL: EEG-TCNet	71.36%	0.795	0.312	65.17%	0.733	0.361	69.76%	0.765	0.325	68.13%	0.752	0.338	64.68%	0.721	0.372
DL: Amin et al., 2019	70.29%	0.772	0.328	64.39%	0.691	0.358	68.25%	0.752	0.336	66.78%	0.744	0.356	63.98%	0.685	0.367
DL: EEGNet	68.82%	0.766	0.330	63.67%	0.658	0.355	67.38%	0.739	0.341	67.19%	0.735	0.344	63.44%	0.654	0.370
DL: Khare and Bajaj, 2021	67.82%	0.751	0.341	63.58%	0.682	0.349	68.05%	0.741	0.348	66.30%	0.739	0.340	62.87%	0.649	0.359
DL: DeepCovNet	67.59%	0.752	0.339	64.56%	0.708	0.342	66.72%	0.745	0.353	65.47%	0.734	0.357	64.23%	0.693	0.363
ML: XGBoost - FS	64.13%	0.698	0.358	59.81%	0.645	0.409	63.04%	0.652	0.372	61.94%	0.639	0.366	58.93%	0.627	0.433
DL: DeePay (shuffled)	50.65%	0.503	0.439	52.08%	0.511	0.450	51.38%	0.516	0.447	51.52%	0.521	0.445	51.71%	0.508	0.457

Accuracy, area under the curve (AUC), and root mean square error (RMSE) for eight different prediction procedures/models, as detailed in the left-most column. We report these scores for five different leave-out procedures, as described in Section “2. Materials and methods”. Results in bold mark the best score in each column.

#### 3.2.3. Dataset size

We also wanted to examine the general effect of the number of subjects on the model’s performance, and whether we reached an optimal number of subjects or could benefit by adding more subjects. Therefore, we investigated the relationship between the total accuracy and the number of subjects used in the training set, using the same procedures as described in Section “2.7. Training and evaluation.” We trained the model on different number of subjects, starting from only 10 subjects and increasing by steps of 10 until reaching the maximum number of subjects, while cross validating each number 10 times and keeping the training set at the same relative size (10%). The results in Appendix B show that the prediction accuracy increased as a function of the number of subjects in the training set. This increase was best explained by a logarithmic function, which showed that the accuracy performance is starting to plateau near the maximal number of subjects, since when adding the last 40 subjects, the accuracy almost did not change (74−75%). However, based on this fitting procedure, it appeared that more subjects would improve the model’s accuracy even further. However, as in any prediction task, we must balance the prediction accuracy we want to achieve with the cost and time of collecting a larger dataset.

### 3.3. Network interpretability

#### 3.3.1. Spectral filters and spatial filters

**FIGURE 5:**
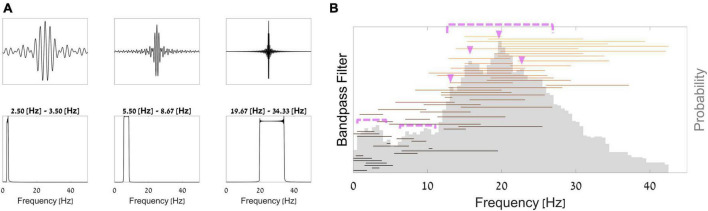
Bandpass filters. **(A)** Top–3 examples of filters from the trained network; bottom–the Fourier transform of each filter. **(B)** Aggregation of all sinc bandpass filters from the network. Each horizontal line represents a single bandpass filter, such that the line starts and ends at the filter’s cut-off frequencies. The histogram in opaque gray shows the overall proportion of each frequency across all filters.

Thanks to the network’s architecture, we were able to interpret the first layer as bandpass frequency filters and the following convolution as band-specific spatial filters. Thus, we can observe the network’s learned parameters in these layers and the process in which it extracted information. From these observations, we can learn what neural information did the network deem most predictive of WTP. In Figure 5A, we plotted 3 example sinc functions that resulted from parameters in the first layer of the network. Below them, we show the results of an FFT analysis for these filters, exemplifying the frequency band that was passed in each of these filters. To understand which frequency bands the network utilized most for prediction, Figure 5B shows all bandpass frequency ranges that the network provided (horizontal lines) and the overall probability of each frequency (gray histogram). As can be seen in Figure 5B, we found that frequencies most prevalent in the first filters of the layer of the trained network (pink triangles and dotted pink lines) were within the beta band 12−30 Hz, peaking at 14 Hz, 17−18 Hz, 20 Hz, and 23 Hz. Additionally, several additional smaller peaks can be found in the delta band (at 2−4 Hz) and the alpha band (at 8−12 Hz).

**FIGURE 6:**
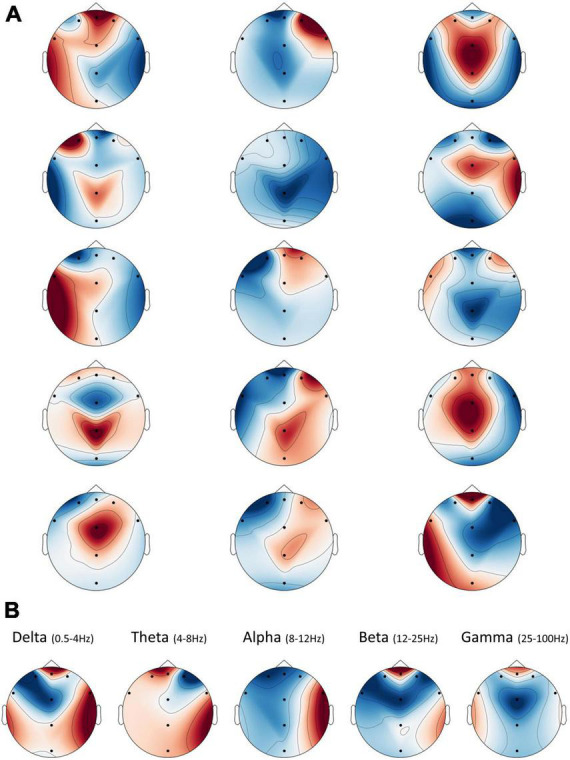
Spatial frequency illustration. The interpolated spatial distribution of weights from the second layer plotted as topographic maps. Note that artifacts appeared in areas further away from the existing electrodes, due to limitations on the interpolation procedure, and should be ignored. **(A)** There are five spatial filters for each sinc bandpass filter, shown as five rows of maps in the figure. Each column represents a corresponding sinc filter, from left to right, as shown in Figure 5A. **(B)** The average spatial weights per frequency band. Spatial weights from the second layer of the network were divided into their respective band (or nearest band), according to the bandpass filter to which they belong from the previous layer. Then, all spatial weights averaged within each band to yield eight final weights per frequency band.

In addition to learning which frequency bands were the most informative for the networks’ predictions, each bandpass filter generated by the network has five unique convolutional “spatial” filters that integrate the information from the different electrodes for each time point separately. We plotted these weights on topographic maps to illustrate how each spectral map, resulting from a bandpass filter, could be combined in various ways with respect to the electrode dimension. However, since our electrode layout did not densely cover the scalp, the interpolations that yielded these maps produced some artifacts in areas further away from our eight electrodes. We urge readers to ignore these areas and focus only on those proximal to the existing electrodes. Figure 6A shows these five maps for the three bandpass filters shown in Figure 5A. They show that while some maps may be redundant and include similar weights (like in column number 3–row 1 and 4, or in column 2–row 4 and 5), others may represent varied and even opposite integrations (as in column 1).

Thus, as in Figure 6B, we opted for an overall interpretation of these filters, by averaging the spatial weights within each of the standard EEG frequency band ranges. These demonstrated that, on average, the network utilized information from the frontal electrode FPZ the most, in all bands but Alpha. We could also see a substantial positive contribution to prediction in frontal Delta, Beta, and Gamma. Meanwhile, the Alpha band showed hemispheric asymmetry in the averaged weights, such that the right hemisphere was positively related to a positive prediction, while the left was negatively related.

#### 3.3.2. Attention layer

**FIGURE 7:**
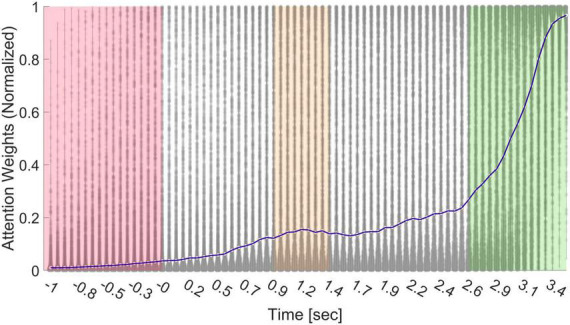
Distribution of weights from the attention layer. When the input reaches the attention layer, it is comprised of 75 time points (due to average pooling), each with its individual weight that is generated per input. We passed all samples through the final network and saved all weights. Thereon, we plotted a violin distribution of all weights per time point to generate this figure. Between time points –1 to 0 s (red area), subjects watched a black screen, between 0.9–1.3 s from stimulus onset (orange area), there was a slight increase in importance, and from time point 2.5 onward, we can observe a substantial increase in importance (green area).

The attention layer provides a weight for each time point in the original EEG signal. These weights can be interpreted as the “importance” of each time point to the following prediction. Figure 7 shows the distribution of weights, normalized between zero and one for better visualization, across all subjects and trials, and their median (represented as the blue line). Between time points −1 to 0 s (red area), subjects watched a black screen (the ITI); therefore, the importance is low for these time points. In time points 0.9−1.3 s from stimulus onset (orange area), we can see an increase in importance, likely signifying initial processing of the stimulus’s value.

From time point 2.5 s to 3.5 s, we can observe a substantial increase in importance (green area). This suggested that the most predictive information appeared after internal deliberation and evaluation of the stimulus (the last second of stimulus observation) and right before the execution of the decision was made possible (when the stimulus was replaced by the BDM scale). However, importantly, even after we removed the final second from analysis, the model could still retain results which are much higher than chance, although losing some accuracy (from 75.09 to 67.15%, Appendix C). This reduction strengthens our claim that some of the predictive information was present in the final second before subjects could start making their decision but plenty of value information was also present before the last second of product observation. However, the reduction in accuracy after removing the last second could not directly attest to the reason why the final second had predictive power. This could be either because of motor preparation, value information, or both.

Therefore, in order to further strengthen the notion that the final second of product observation contributed to prediction because it contained value information and not a motor preparation signal, we conducted an additional analysis on subjects’ reaction times (RTs). We calculated the cumulative distribution of RTs over all trials (see Appendix D). We can see that in 94% of the trials, subjects responded more than 1 s after the end of the observation phase, and in almost 60% of the trials, subjects responded more than 2 s after the end of the observation phase. Previous studies had shown that the signal for motor preparation, as evident in the EEG, appears between 0.25 s to 0.6 s, on average, before motor execution (Georgopoulos et al., 1989; Bai et al., 2011; Lew et al., 2012). In our study, most of subjects’ decisions started a lot longer (1−2 s), which extremely reduces the chances that a motor preparation signal contributed to the predictive information of our model.

### 3.4. Standard EEG analysis

#### 3.4.1. ERP results

**FIGURE 8:**
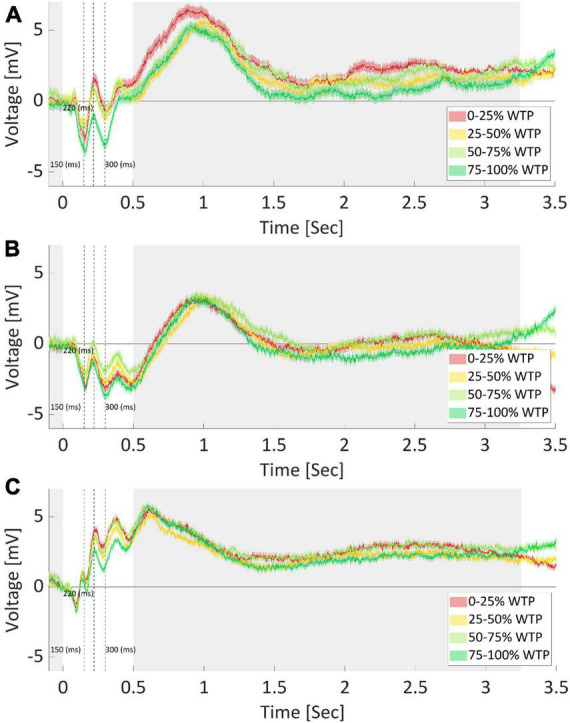
Average ERP per quartile of WTP values. EEG recordings of electrode FPz **(A)**, F7 **(B)**, and Cz **(C)**, after averaging for each WTP quartile, across subjects and products. The product image appeared at time zero, marking the start of a trial. Opaque bounds around a line signify the SEM at each data point. Cleary, each electrode recorded different responses and required diverse feature extraction procedures, further demonstrating the need for optimized and automatic feature extraction. The shaded areas mark time points where no difference between WTP quartiles was found. Dotted lines indicate the 150, 220, and 300-ms mark, respectively.

As can be seen in Figure 8A, three components of interest emerged. The N150 (negative potential after 150 ms), P220 (positive potential after 220 ms), and N300 (negative potential after 300 ms) showed varying amplitudes across the WTP quartiles. The N150 has a lower amplitude for the highest and lowest WTP quartiles, perhaps measuring a response to the saliency of the products. We averaged the signal between 140 and 160 ms per sample and found a significant difference between quartiles for this component [*F*(3) = 5.12, *p* < 0.001, one-way ANOVA]. We conducted *post hoc* multiple comparison testing (Tukey), which revealed that the highest quartile had significantly lower N150 compared to the first (*p* < 0.001) and second (*p* < 0.05) quartiles. The P220 and N300 components, in contrast, seem to exhibit sensitivity to value by having the lowest amplitude for the highest WTP quartile, perhaps encoding valuations. Again, we averaged the signal between 210 to 230 ms and 290 to 310 ms and found a significant difference between quartiles for these components as well [P220: *F*(3) = 10.12, *p* < 0.001; N300: *F*(3) = 9.58, *p* < 0.001, one way ANOVA]. The same *post hoc* analysis revealed that the 4th quartile was significantly lower than all other quartiles in both components (*p* < 0.001 in all cases). We also broke up the analysis and examined the activity for every 0.5 s, starting at 0.5 s from stimulus onset, up to 3.5 s from the onset. However, these time points did not yield significant results.

However, note that many other characteristics of the signal could be different between the quartiles, in addition to what we captured by the three components identified (N150, P220, and N300). First, we demonstrated in the Section “3.2.1. WTP prediction” that predictions based on manually extracted features, including these components, were inferior to the automatic extraction by the neural network, aiding the conclusion that there were likely additional value-related signal characteristics that the DLN found than those captured by these components. For instance, as is common in the field, we manually and arbitrarily (by “visually inspecting the data”) decided in which time window to average the signal and on which to conduct our statistical analyses. There are endless possibilities for manually choosing the onset and offset time points, and the window size for the analysis. It is very hard to know, *a priori*, what would be the best combination of time window parameters to use. Moreover, any post-decision could lead to an inflated type one error and over fitting the data. To further highlight this problem, we repeated the exact same analysis for electrodes F7 and Cz (see Figures 8B, C, respectively). Even without explicitly conducting a formal statistical analysis, it is clear that in these electrodes, the ordering of the quartiles is different for the three ERP components mentioned above. It might be that we could find in electrodes F7 and Cz other components, which would be significantly different between the WTP quartiles in a meaningful manner (either ascending or descending as a function of WTP values). However, finding these components could mean that we would need to examine endless combinations of time window parameters. This further strengthens the need for automatic feature extraction that would be sensitive to various time points, window sizes, and electrodes, as we incorporated in our deep learning network. We suggest that this manual procedure is most likely sub-optimal for prediction compared to deep learning.

#### 3.4.2. Spectral analysis

**FIGURE 9:**
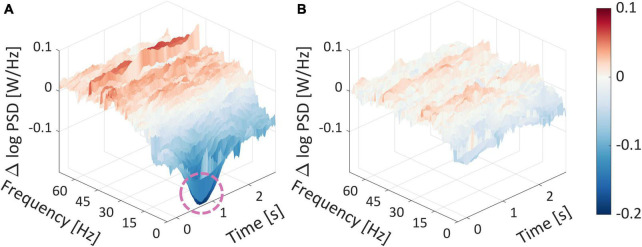
Spectrogram differences for WTP quartiles. **(A)** The difference between the average spectrogram of the top and bottom average spectrogram quartiles. **(B)** The difference between the average spectrogram of the mid-high and mid-low average spectrogram quartiles. The product image appeared at time zero, marking the start of a trial. The dashed pink circle surrounded a substantial difference in the log PSDs.

As can be seen in Figure 9A, there is a substantial difference between the top and bottom average spectrograms at the lower frequency range (0.5−10 Hz), around 0.5 s after the stimulus has appeared. This is indicated by the pink circle around a large dark blue dip at that time point, which reached a nearly −0.2 difference in log PSD. Importantly, additional frequencies at varied time points differed between high and low WTP values, as indicated by the dark red and blue colors, which represent a log difference of nearly 0.1 and −0.2 PSD, respectively.

The fact that there are numerous time points and frequencies that are significantly different between the top and bottom quartiles strengthens our notion that it would be futile to attempt to identify the most statistically relevant time points and frequencies, which would undoubtedly result in false positives even after correction for multiple comparisons. This suggests that the subjective value represented in the frequency domain is very complex. Trying to quantify subjective value representation into several features with any feature selection technique would probably be suboptimal and less generalizable. This serves as another testament as to why we advocate extracting features from the EEG signal automatically. The manual alternative, laboriously handpicking specific frequencies and time points, or aggregating frequency ranges to frequency bands (Alpha, Delta, etc.) or across time points, is suboptimal and involves information loss, as we demonstrated in the previous section based on the network prediction results.

## 4. Discussion

In this study, we showed that using a state-of-the-art DLN, it is possible to predict subjects’ WTP values of products based on EEG recorded from the same subjects while they observed an image of the product. The unique network architecture, specially geared for EEG decoding and interpretation, alongside the meta-processes we used, reached 75.09% accuracy predicting above and below the median WTP value, with 0.832 AUC. The network also achieved 0.276 RMSE when predicting the continuous WTP values. In all cases, our network was better than other deep learning networks, exceeded results from a machine learning approach that utilized manual feature extraction, and surpassed random results (shuffled labels) by a large margin.

Moreover, we showed that our model was able to generalize over products, subjects, and categories, through the various leave-out procedures we conducted. Mostly, we found that our model was able to maintain competitive results for a more difficult, applicable, and generalizable setting–1-category-out and 20-subjects-out. This result may especially interest practitioners, who hope to apply the model on both subjects and products that the model had not trained on. Furthermore, the generalization over categories was particularly important, since some categories included more higher-valued items on average, while others were more homogenous with lower-value items. This could confound our results with an alternative interpretation, that the model identified different categories rather than high/low valuations. However, the 1-category-out results showed that removing an entire category, be it one containing lower valued items on average, or another containing higher valued items, did maintain near-maximal performance, while reducing it by a small amount (from 75.09 to 71.44%). It is unlikely, therefore, that the model identified information related to the category in the EEG signal, which is by itself a higher-level and more abstract concept to extract from EEG. Nonetheless, the reduction in performance when comparing the 1-category-out analysis to our general analysis using all products could suggest that some information related to the category of products was used. However, a different explanation could be that prediction of valuations from a new category, which the model was not trained on, was more difficult. Another possible reason for this reduction is that removing a high-valued category, or a low-valued category, reduces the disparity between lowest and highest valued items in the dataset, making it more difficult for the model to differentiate between valuations. Regardless of these possible reasons, which could all be true at once, this examination further emphasizes the need for multiple categories and products in the data, with highly varied valuations for each, to make sure that models are able to identify value-related information rather than stimulus-related information (such as product identity, category, and so on).

Our results strengthen our notion that a DLN framework approach, where the network itself determined the most relevant features and their appropriate weightings, was better than an approach where one must determine, *a priori*, the exact features to input the model. Because there are endless possibilities and degrees of freedom to this process, we strongly suggest moving beyond manual feature extraction toward automated feature extraction based on deep learning approaches.

Importantly, we showed that prediction scores were dependent on the extremity of WTP values. That is, prediction scores were superior for the prediction of the most extreme WTP values (0−20%/80−100%) and decreased as WTP values became closer to each other. This reflects the notion of “neural distance” that was also found in previous studies, which states that as the distance between subjective values increase, so does their neural representation, and prediction becomes better (Levy et al., 2011; Telpaz et al., 2015; Hakim et al., 2021).

We conducted several vital analyses of the behavioral results to ensure that the WTP values elicited by the BDM task were a close proxy to the subjective values that subjects had for the products, and that we predicted these values and not product identity. First, across subjects, there was a wide distribution of bids for each product, indicating a variety of valuations on the offered products between subjects. Second, we used a large number of products (Cecotti et al., 2014), which further reduced the probability that the network could identify neural information related to the product stimuli when predicting WTP, as WTP values would have to align similarly across all products for all subjects. Third, attempting to predict WTP based on product identities resulted in a prediction accuracy that was only slightly above shuffled data. Lastly, subjects demonstrated broadly consistent bids for each product across blocks, as demonstrated by high correlations between the bids across each pair of blocks. This suggests that subjects’ bids were stable, and therefore, we could use them as a reliable representation of subjective value, and hence, as stable prediction labels.

We also analyzed our EEG data using standard approaches commonly used in the literature. We manually extracted features from specific time points and pre-defined frequency bands to look for components that could help predict WTP values. We wanted to show that this manual feature extraction and inspection, while it may lead to insightful observations, is very arbitrary and yields suboptimal predictions.

First, we conducted an event-related analysis. Based on this analysis, we found several interesting time points in the EEG time-series, averaged across all subjects and trials for each quartile of WTP. The average signal for the highest WTP quartile was lower than other quartiles on the 150-ms negative peak, 220-ms positive peak, and 300-ms negative peak. Event-related components within this time range were found to be related to value in various studies, such as the N200 (Folstein and Van Petten, 2007; Telpaz et al., 2015; Gajewski et al., 2016; Goto et al., 2017). Finally, the average signal of the lowest quartile of WTP values showed the highest increase in voltage after the 300-ms mark, which could be related to the P300 component, that has been shown to be involved in valuation and decision making (Miltner et al., 1997; Gehring and Willoughby, 2002; Holroyd and Coles, 2002; Yeung and Sanfey, 2004; Hajcak et al., 2005, 2007; Sato et al., 2005; Toyomaki and Murohashi, 2005; Yeung et al., 2005; Luu et al., 2009; Holroyd et al., 2011; Proudfit, 2015; Mushtaq et al., 2016). Importantly, by visually inspecting the EEG times-series, averaged by WTP quartiles, we showed that different electrodes produced various components with diverse relationships to value.

The multitude of optional components to choose from, which depend on varying scalp locations and varying time points, makes it very unlikely that a manual approach to feature extraction would allow for an accurate and generalizable model. Critically, we could not have known in advance to take these precise components, as we only found them through “peeking” into our prediction labels. This means that even if these features would contribute to WTP prediction, as they likely would in our case (since they fitted our data), they would not necessarily be found as the most contributive in another dataset. Moreover, when we added these components for the prediction of WTP using ML models, it still resulted in far worse predictions than the DLN approaches. Hence, the large variety of optional ERP components, the lack of explicit directions on how to choose among them, and the inferior results from the ML prediction approach using manual extraction further substantiate our claim that automatic feature extraction should surpass any attempt to capture value-related information hidden in the EEG signal manually.

In addition to maximizing our deep learning network’s prediction accuracy, we also engineered it so that we could interpret the results, and therefore, learn from it regarding the neural representation of value. Based on examining the output of the first and second layer of the network, we found evidence that some EEG features previously shown to be related to prediction of subjective value also contributed to prediction in our network. Specifically, based on the output of the network’s first layer, we learned that the beta band was most important for the prediction of WTP, in correspondence with previous research (Braeutigam et al., 2004; Boksem and Smidts, 2015), while alpha and delta bands also contributed substantially (Braeutigam et al., 2004; Smith and Gevins, 2004; Astolfi et al., 2008; Khushaba et al., 2013; Ravaja et al., 2013). Besides, by averaging the spatial weights from the second layer within each of the standard frequency bands, we found that the network relied mainly on the frontal electrode FPZ for its prediction, in correspondence with previous research which related frontal cortical activity with value (Hakim and Levy, 2019). Meanwhile, we found evidence for hemispheric asymmetry in the alpha band, such that the right hemisphere was positively related to a positive prediction, while the left was negatively related. This EEG measure is commonly considered as a measure of engagement, or approach/avoidance (Davidson, 1998; Sutton and Davidson, 2000; Ohme et al., 2009, 2010; Vecchiato et al., 2010, 2011; Koelstra et al., 2012; Ravaja et al., 2013; Venkatraman et al., 2015; Cartocci et al., 2016; Ramsøy et al., 2018). Another EEG measure inspected in our previous paper (Hakim et al., 2021), “Inter-Subject Correlation,” is a measure of similarity between subjects, and therefore, we could not find means to relate it to the results in the current study. However, comparing the results from the individual spectral and spatial filters found in our study to EEG results found in previous literature would be amiss. We can see that out of all filters that the network generated, some frequencies and electrodes correspond with results from previous studies, while others do not. This provides further evidence that, while literature-based manual feature extraction could provide predictive information and meaningful insights, there could still be vast possible features that contain critical information that researchers could be unable to find through manual exploration.

Moreover, the frequency bands we identified in the first layer and the scalp distribution of each of the spectral maps from the second layer were both highly varied. As in the case of the event-related analysis, this demonstrated that it is near impossible to manually identify the most predictive frequencies and their most fitting scalp distribution. Unfortunately, this is often the approach in many EEG studies. It is very unlikely that researchers could estimate which frequencies to extract and how to combine their scalp information. We propose that using a DLN framework approach provides an automated and better solution to address this. Moreover, studies often combine information from electrodes in a single manner, such as averaging or conducting an Independent Component Analysis (ICA), and combine the electrodes for all frequency ranges using only one method (Luck, 2014). In contrast, our network architecture defined several unique electrode combinations for each bandpass filter separately, enabling substantially larger expressiveness in extracting features from the signal. The network produced several sets of weights for the electrode dimensions for each spectral map produced by a bandpass filter. Some were unique while others were redundant, thus enabling varied ways to combine the electrodes’ information that were specialized and independent per bandpass filter, without the need for relying on the popular frequency bands (alpha, beta, gamma, delta, and theta).

Finally, the attention layer was able to expose the time points that were most important for WTP prediction. Averaging the attention weights across all samples revealed an uplift in predictive information 1 s after stimulus onset. This possibly reflected early processing of the stimulus’s value without converging to a final valuation yet. Thereon, the averaged weights increased substantially until reaching their highest just before decision execution. Moreover, removing the last second from the analysis still resulted in a robust predictive model, although with slightly diminished performance likely due to losing important value-related predictive information present in the last second. The sharp increase in importance toward the end of stimulus observation might be interpreted as motor preparation toward moving the mouse cursor to either the left or right of the scale–and not as value information. However, we do not think this is the case because we randomized the initial location of the marker on the scale, so subjects could not have known in advance whether and by how much they would need to move the mouse cursor to the left (low value) or the right (high value) to reach their desired bid. Hence, there might be a general motor preparation signal in the EEG, but it cannot be correlated with the actual value (WTP bid) subjects were planning to mark on the scale. In addition, the response time analysis showed that most subjects took more than 2 s to respond after stimulus offset. This time frame is far beyond the scope of the motor preparation time frame evident in the EEG signal, as previous studies showed (Georgopoulos et al., 1989; Bai et al., 2011; Lew et al., 2012). Therefore, the predictive information hidden in the EEG signal from the last second of product observation cannot be attributed to a motor preparation signal for moving the value scale. We propose that the rise of importance toward decision execution demonstrates that subjects thought about and determined their bid gradually while they watched the product, and that it peaked just before they had to report it. This process resembles the well-known evidence accumulation models for describing choice (Ratcliff, 1978; Gold and Shadlen, 2007; Krajbich et al., 2010).

In this study, we tackled several of the difficulties in EEG modeling mentioned in the In this study, we tackled several of the difficulties in EEG modeling mentioned in the introduction. First, to address the small sizes of EEG datasets, we created a large new dataset in our lab, with more than 200 subjects and with 216 trials per subject. Moreover, we used neural network layers that are compact, efficient, and low in parameters, that can accommodate smaller datasets. Second, to avoid the elaborate manual feature extraction often required in EEG studies, we fed the full EEG data into the DLN without almost any preprocessing, artifact rejections or filtering, and designed its layers to perform all feature extraction “automatically” through training. These layers essentially replaced the standard practice of (to our opinion arbitrary) selection of the “classic” frequency bandpass filters (alpha, beta, gamma, delta, and theta). Moreover, some of the layers served as spatial filters that identified the optimal weights across the electrodes, and other layers identified important components in the time domain (Bi-LSTM and attention) instead of arbitrarily defining the time window for analysis. Third, regarding the high dimensionality of EEG data, we used a small number of electrodes to reduce this dimensionality and demonstrate that prediction can still be accomplished when using low density electrode arrays. Next, to handle the intrinsic noise of EEG data, the summation layers decrease the dimensionality of the data substantially, facilitating noise reduction by forcing compression. Lastly, the variation in the EEG’s signal between subjects using neural network approaches is theoretically difficult to tackle. Although our DLN does not include specific means to handle this, we were able to generalize across subjects, with our results on subject-out prediction. A better solution to this issue would be a great opportunity for future work.

There are several avenues of research that could expand our study. First, our predictions were limited to valuations elicited immediately after recording the EEG response, but future studies could examine the ability to predict lasting valuations elicited an hour or a week after EEG was recorded. The consumer neuroscience field and the neuromarketing industry are interested in predicting durable and stable subjective values, so this study can provide the basis for researchers to examine to what degree these long-term valuations can be predicted. Also, researchers may want to examine other methods for preference elicitation and not WTP, such as binary choices. Additionally, marketers often desire to predict a product’s success in the general population rather than predicting individual subjective valuations. Future research could also inspect whether EEG recordings can predict valuations on products in the general population, as estimated via general sales, YouTube measures, or other accepted metrics. Finally, our study was limited to valuations over products introduced via product images. Researchers could build upon these findings and examine whether they could predict valuations of products from EEG recorded while subjects observe real marketing material, such as ad images or commercials. Demonstrating that WTP could still be predicted based on more complex and ecological stimuli would provide neuromarketers with valuable evidence of the EEG signal’s capability as a tool to assess ad effectiveness and predict marketing gains.

## 5. Conclusion

We were able to show that a deep-learning approach with carefully constructed architecture could be ideal for EEG-based prediction of WTP, surpassing both manual approaches and generic DLN frameworks. Our DLN generated features to a degree of complexity, spatially, and spectrally that could not have been achieved through manual inspection and feature extraction. Importantly, our data-driven approach shed light on some aspects of the valuations process, as reflected in the EEG signal, that were not previously investigated or were unlikely to be included in a prediction model that uses manual feature extraction. The network employed an abundance of frequencies, spatial distributions, and time points, focusing mainly on the aftermost time points. Thus, neuromarketing practitioners can utilize our DLN framework to enhance their attempts to predict WTP based on EEG data, albeit on real marketing stimuli or using long-term preference elicitation methods. Meanwhile, EEG and decision-making researchers can employ this framework to learn more about the valuation process by investigating the network’s resulting parameters and exploring which neural features the network deemed most essential for prediction.

## Data availability statement

The original contributions presented in this study are included in the article/Supplementary material, further inquiries can be directed to the corresponding author.

## Ethics statement

The studies involving human participants were reviewed and approved by the Ethics Committee of Tel Aviv University. The patients/participants provided their written informed consent to participate in this study.

## Author contributions

AH performed the data analysis and visualizations, oversaw the data collection, and created the prediction models. IG contributed to the modeling, designed the model optimizations, and wrote the related sections. AH and DL designed the experiment and wrote the manuscript. SY conducted the experimental procedure. All authors read and approved the final manuscript.
