# Supplementary material for: DeePay: deep learning decodes EEG to predict consumer’s willingness to pay for neuromarketing
Source: Front Hum Neurosci. 2023 Jun 5;17:1153413. doi: 10.3389/fnhum.2023.1153413 (PMC10277553; doi:10.3389/fnhum.2023.1153413)
Supplement: Supplementary file 1 [file Data_Sheet_1.DOCX]

APPENDIX A

LIST OF PRODUCTS

| ***Category*** | ***Products*** | | | | | |
| --- | --- | --- | --- | --- | --- | --- |
| ***Appliances*** | Toaster | Kettle | Iron | Heat Blower | Coffee Maker | Portable Battery |
|  | (66 NIS) | (56 NIS) | (65 NIS) | (66 NIS) | (69 NIS) | (60 NIS) |
|  | Kitchen Scale | Wireless Mouse | Keyboard | Non-stick Pan | Hotdog Cooker | Ceramic Stove |
|  | (50 NIS) | (59 NIS) | (59 NIS) | (85 NIS) | (85 NIS) | (83 NIS) |
| ***Care*** | Women Mascara | Men Gillette Razor | Men care package - Crema | Men care package - Nivea | Men Deodorant | Men Perfume |
|  | (65 NIS) | (64.9 NIS) | (45 NIS) | (54.9 NIS) | (69 NIS) | (89 NIS) |
|  | Men Face Soap | Electric Toothbrush | Women Makeup | Hair blower | Women Hair Straightener | Women Daycream |
|  | (70 NIS) | (99 NIS) | (99 NIS) | (89 NIS) | (99 NIS) | (59.9 NIS) |
| ***Experience*** | Stand-up show tickets | Cooking workshop with a private chef | Two tickets for the theater - Habima | Voucher for a cocktail workshop | Drumming lesson | Surfing lesson |
|  | (65 NIS) | (89 NIS) | (69 NIS) | (69 NIS) | (59 NIS) | (97 NIS) |
|  | Couple’s breakfast | Reflexology treatment | Massage treatment | Cinema tickets | Ticket for a hot air balloon flight | Segway experience |
|  | (79 NIS) | (89 NIS) | (99 NIS) | (88 NIS) | (64 NIS) | (89 NIS) |
| ***Food*** | Burger meal | 6 Bottles of Coke Zero | Wine bottle | Grey Goose vodka bottle | Weihenstephan beer - 6 pack | Praline case |
|  | (99 NIS) | (45.5 NIS) | (59.9 NIS) | (99 NIS) | (79.2 NIS) | (54 NIS) |
|  | Sweet pastries | Whiskey bottle | Mentos pack | 12 Packs of Pringles | Domino's Pizza tray | Kinder package |
|  | (56 NIS) | (79 NIS) | (91.26 NIS) | (48.2 NIS) | (71 NIS) | (49.26 NIS) |
| ***Office*** | Parker Pen | Calculator | Sticky bulletin board | Newton Cradle | Stapler | Pointer |
|  | (84.9 NIS) | (59 NIS) | (64.9 NIS) | (89 NIS) | (96.64 NIS) | (50 NIS) |
|  | Photo printing paper | Gold USB | Chequered Paper | Stormtrooper USB | Book safe | Laptop case |
|  | (59 NIS) | (78 NIS) | (49.9 NIS) | (69 NIS) | (99 NIS) | (99 NIS) |
| ***Gambles*** | 75% to win 70 | 50% to win 100 | 25% to win 200 | 75% to win 80 | 50% to win 120 | 25% to win 240 |
|  | (50 NIS) | (50 NIS) | (50 NIS) | (60 NIS) | (60 NIS) | (60 NIS) |
|  | 75% to win 95 | 50% to win 140 | 25% to win 280 | 75% to win 106 | 50% to win 160 | 25% to win 320 |
|  | (70 NIS) | (70 NIS) | (70 NIS) | (80 NIS) | (80 NIS) | (80 NIS) |

**Appendix A. List of Products**. The table includes names of all products, divided into their respective categories. The real-world prices appear in brackets. Gambles detail the expected value in brackets.
