# Supplementary material for: DeePay: deep learning decodes EEG to predict consumer’s willingness to pay for neuromarketing
Source: Front Hum Neurosci. 2023 Jun 5;17:1153413. doi: 10.3389/fnhum.2023.1153413 (PMC10277553; doi:10.3389/fnhum.2023.1153413)
Supplement: Supplementary file 2 [file Data_Sheet_2.DOCX]

APPENDIX B

Prediction Accuracy by Number of Subjects

**
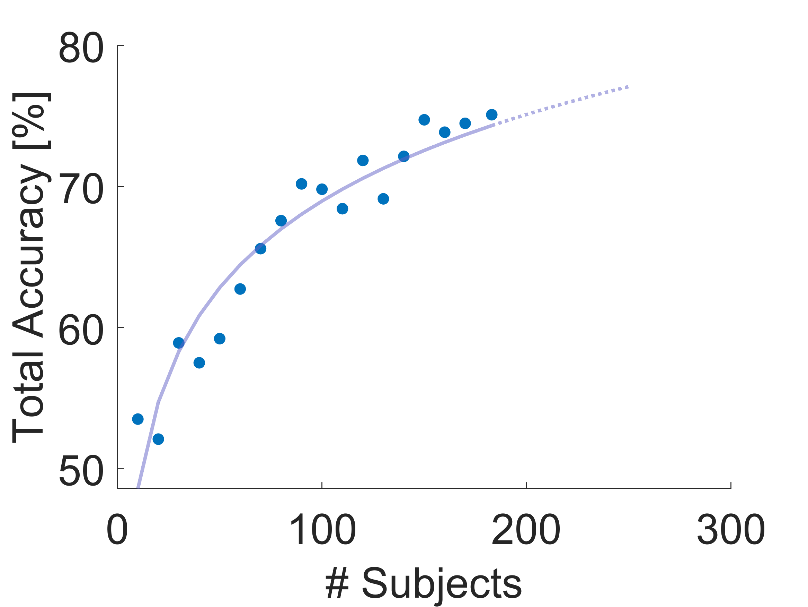
**

**Appendix B. Prediction Accuracy by Number of Subjects**. The y-axis shows the total accuracy of our main model (DeePay), in percentages, calculated as described in the methods section. The scattered dots display the accuracy obtained through training the model on different amounts of subjects, from 10 to 183, in steps of 10. The continuous line represented the fit of these dots to a simple logarithmic function [f(x)=a+b*log(x]), while the dashed line shows extrapolation of the function to additional hypothetical amounts of subjects, up to 250.
