# Supplementary material for: DeePay: deep learning decodes EEG to predict consumer’s willingness to pay for neuromarketing
Source: Front Hum Neurosci. 2023 Jun 5;17:1153413. doi: 10.3389/fnhum.2023.1153413 (PMC10277553; doi:10.3389/fnhum.2023.1153413)
Supplement: Supplementary file 3 [file Data_Sheet_3.DOCX]

APPENDIX C

Prediction Results with Last Second Excluded

| **Timepoint Excluded** | **Last Second** | | |
| --- | --- | --- | --- |
| **Score** | Acc | AUC | RMSE |
| DL: DeePay | **67.15%** | **0.741** | **0.343** |
| DL: EEG-TCNet | 64.53% | 0.680 | 0.362 |
| DL: EEGNet | 62.92% | 0.656 | 0.368 |
| DL: DeepCovNet | 62.05% | 0.677 | 0.385 |
| ML: XGBoost - FS | 59.78% | 0.675 | 0.412 |
| DL:DeePay (Shuffled) | 50.32% | 0.504 | 0.458 |

**Appendix C. Prediction Results with Last Second Excluded**. The table shows the prediction results, obtained through the same procedures as in table 1 in the manuscript, only excluding the last second of product observation (2.5s-3.5s). Results are shown only for the most successful models from the main analysis in table 1.
