# Supplementary material for: DeePay: deep learning decodes EEG to predict consumer’s willingness to pay for neuromarketing
Source: Front Hum Neurosci. 2023 Jun 5;17:1153413. doi: 10.3389/fnhum.2023.1153413 (PMC10277553; doi:10.3389/fnhum.2023.1153413)
Supplement: Supplementary file 4 [file Data_Sheet_4.DOCX]

APPENDIX D

Cumulative Distribution of Response Times


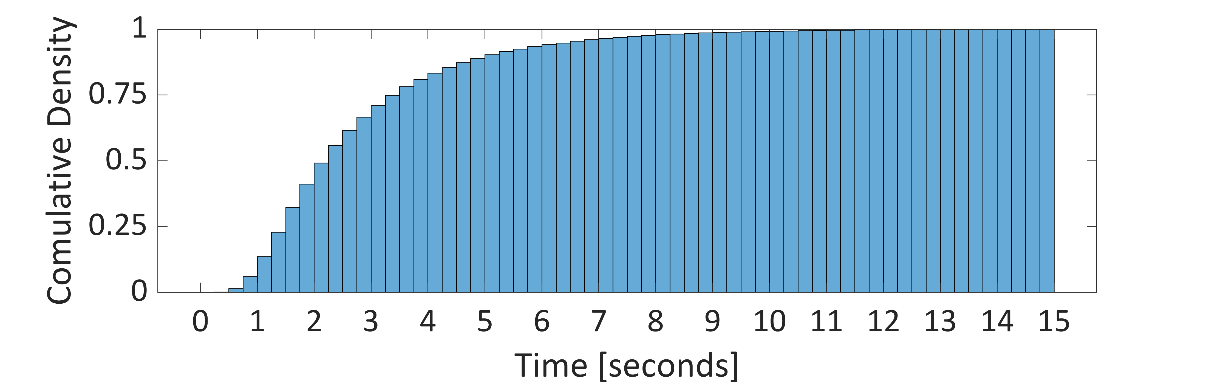


**Appendix D. Cumulative Distribution of Response Times**. After the 3.5 second of product observation had passed, subjected had 15 seconds to respond with their bid on the product. This graph shows the cumulative distribution of the response time, in intervals of 0.25 second, from zero to 15 second. The y-axis shows the fraction of total trials that had response times at the corresponding timepoint in the x-axis and beforehand (cumulative).
