# Supplementary material for: DeePay: deep learning decodes EEG to predict consumer’s willingness to pay for neuromarketing
Source: Front Hum Neurosci. 2023 Jun 5;17:1153413. doi: 10.3389/fnhum.2023.1153413 (PMC10277553; doi:10.3389/fnhum.2023.1153413)
Supplement: Supplementary file 5 [file Data_Sheet_5.DOCX]

APPENDIX E

Confusion Matrix and Report for Main Results


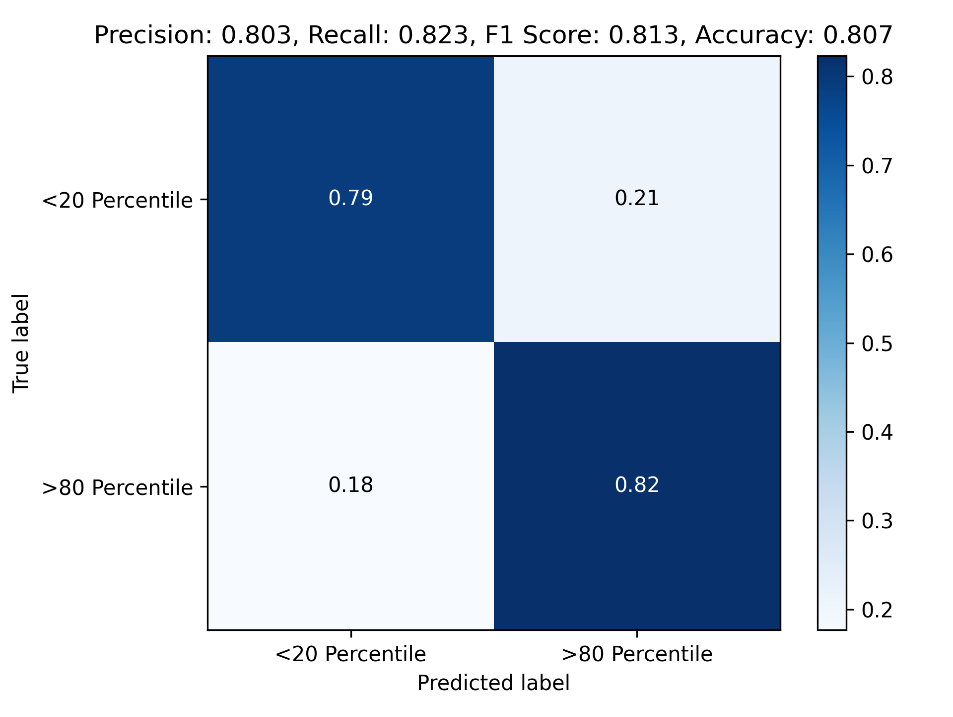


**Appendix E. Confusion Matrix and Report for Main Results**. The complete confusion matrix, along with precision, recall, F1 score and accuracy, for our main test results – prediction of low (0-20%) versus high (80-100%) quantiles, with DeePay.
